# Comparison of Anti–Programmed Cell Death Ligand 1 Therapy Combinations vs Sunitinib for Metastatic Renal Cell Carcinoma: A Meta-analysis

**DOI:** 10.1001/jamanetworkopen.2023.14144

**Published:** 2023-05-18

**Authors:** Brigida Anna Maiorano, Davide Ciardiello, Evaristo Maiello, Giandomenico Roviello

**Affiliations:** 1 Oncology Unit, Istituto di Ricovero e Cura a Carattere Scientifico (IRCCS) Foundation Casa Sollievo della Sofferenza, San Giovanni Rotondo, Italy; 2 Department of Precision Medicine, Luigi Vanvitelli University of Campania, Naples, Italy; 3 Division of Gastrointestinal Medical Oncology and Neuroendocrine Tumors, European Institute of Oncology, IRCCS, Milan, Italy; 4 Department of Health Sciences, Section of Clinical Pharmacology and Oncology, University of Florence, Florence, Italy

## Abstract

This meta-analysis of 3 randomized clinical trials evaluates the association of combined anti–PD-L1 agents with survival and response rate among patients with metastatic renal cell carcinoma.

## Introduction

In the past 10 years, immune checkpoint inhibitors (ICIs) have been used to manage metastatic renal cell carcinoma (mRCC). Different combinations of ICIs and tyrosine kinase inhibitors or double ICIs have been approved by regulatory agencies for first-line treatment of mRCC after demonstrating prolonged survival vs sunitinib in randomized clinical trials (RCTs). From a pharmacological perspective, anti–programmed cell death 1 (PD-1) and anti–programmed cell death ligand 1 (PD-L1) agents behave similarly. However, combinations based on anti–PD-1 vs anti–PD-L1 agents currently dominate first-line treatments of mRCC. We aimed to assess the association of anti–PD-L1 treatment combinations with survival and response rate among patients with mRCC.

## Methods

The literature search for this meta-analysis was conducted in December 2022 and followed the PRISMA reporting guideline. Randomized clinical trial articles on anti–PD-L1 agents as first-line mRCC treatment were selected (eFigure in Supplement 1). We included 3 RCTs^1,2,3^ that assessed overall survival (OS), progression-free survival (PFS), and overall response rate (ORR). For OS and PFS, summary hazard ratios (HRs) were calculated; for ORR, odds ratio (OR) was assessed. Random- or fixed-effects models were used, depending on study heterogeneity. Two-sided *P* < .05 indicated statistical significance. RevMan 5.4 (Cochrane Collaboration) was used for statistical analyses.

## Results

Atezolizumab plus bevacizumab (IMmotion150 and IMmotion151 trials^1,3^) and avelumab plus axitinib (JAVELIN Renal 101 trial^2^) were administered in the intervention groups (n = 1100; 784 males [71.3%], 316 females [28.7%], mean [SD] age, 62 [0.45] years), and sunitinib was administered in the control group (n = 1107; 776 males [77.1%], 231 females [22.9%], mean [SD] age, 61 [0.58] years). Overall, the combination of anti–PD-L1 agents was not associated with a risk of death (HR, 0.88; 95% CI, 0.75-1.03; *P* = .11) but was associated with a reduced risk of progression (HR, 0.78; 95% CI, 0.69-0.88; *P* < .001) compared with sunitinib. Moreover, the trials did not report a higher ORR with anti–PD-L1 agents than sunitinib (OR, 1.63; 95% CI, 0.79-3.35; *P* = .19). In patients with PD-L1 expression, no differences in OS were detected (HR, 0.84; 95% CI, 0.67-1.05; *P* = .12), but PFS (HR, 0.66; 95% CI, 0.56-0.79; *P* < .001) and ORR (OR, 2.28; 95% CI, 1.17-4.46; *P* = .02) were favorable with a combination of anti–PD-L1 agents vs sunitinib (Table).

**Table.** Clinical Trials of Anti–PD-L1 in Metastatic Renal Cell Carcinoma: Data From Single Studies and Pooled Efficacy Data

Trial; source	ITT population				PD-L1 combination			
	No. of patients	Median OS, mo	Median PFS, mo	ORR, %	No. of patients	Median OS, mo	Median PFS, mo	ORR, %
IMmotion151 phase 3; Rini et al,^1^ 2019								
Atezolizumab + bevacizumab	454	33.6	11.2	37.0	178	34.0	11.2	43.0
Sunitinib	461	34.9	8.4	33.0	184	32.7	7.7	35.0
HR (95% CI)		0.93 (0.76-1.14)	0.83 (0.70-0.97)			0.84 (0.62-1.14)	0.74 (0.57-0.96)	
JAVELIN Renal 101 phase 3; Motzer et al,^2^ 2019								
Avelumab + axitinib	442	NE	13.8	51.4	270	NE	13.8	55.2
Sunitinib	444	NE	8.4	25.7	290	NE	7.2	25.5
HR (95% CI)		0.80 (0.62-1.03)	0.69 (0.56-0.85)			0.83 (0.59-1.17)	0.61 (0.47-0.79)	
IMmotion150 phase 2; McDermott et al,^3^ 2018								
Atezolizumab + bevacizumab	101	NA	11.7	43.0	50	NA	14.7	46.0
Sunitinib	101	NA	8.4	29.0	60	NA	7.8	27.0
HR (95% CI)			0.82 (0.59-1.14)				0.99 (0.64-1.53)	
Pooled data								
HR (95% CI)		0.88 (0.75-1.03)	0.78 (0.69-0.88)	OR: 1.63 (95% CI, 0.79-3.35)		0.84 (0.67-1.05)	0.66 (0.56-0.79)	OR: 2.28 (95% CI, 1.17-4.46)
*P* value		.11	<.001	.19		.12	<.001	.02
*I*^2^, %		0	0	92		0	0	82

Abbreviations: HR, hazard ratio; IMmotion150, A Phase II, Randomized Study of Atezolizumab (Anti–PD-L1 Antibody) Administered as Monotherapy or in Combination With Bevacizumab Versus Sunitinib in Patients With Untreated Advanced Renal Cell Carcinoma; IMmotion151, A Study of Atezolizumab in Combination With Bevacizumab Versus Sunitinib in Participants With Untreated Advanced Renal Cell Carcinoma; ITT, intention-to-treat; JAVELIN Renal 101, A Study of Avelumab With Axitinib Versus Sunitinib in Advanced Renal Cell Cancer; NA, not available; NE, not estimable; OR, odds ratio; ORR, overall response rate; OS, overall survival; PD-L1, programmed cell death ligand 1; PFS, progression-free survival.

## Discussion

Anti–PD-L1 and anti–PD-1 agents prevented PD-L1 from interacting with PD-1; however, the 2 classes slightly differed in pharmacokinetics and pharmacodynamics. Because PD-1 is expressed on the T-cell membrane surface, anti–PD-1 agents are more effective than anti–PD-L1 agents in activating T cells even with lower or no PD-L1 expression, which partially explains why ICIs were active in cases with lower or absent PD-L1 expression. In contrast, PD-L1 is expressed by tumor cells; therefore, anti–PD-L1 agents dysregulate tumor cell signaling rather than simply acting on T cells. In other tumor subtypes, such as non–small cell lung cancer, evidence suggests that anti–PD-1 agents could be more effective than anti–PD-L1 agents, as anti–PD-1 agents simultaneously block PD-1 binding with both PD-L1 and PD-L2.^4^ However, anti–PD-L1 agents did not influence the PD-1–PD-L2 interaction, which may inhibit T-cell activation. Therefore, in the setting of anti–PD-L1 agent use, the PD-1 or PD-L2 could be used by the tumor to escape the antitumor immune response.^4^

Further evidence of the lack of effectiveness of anti–PD-L1 agents in RCC comes from the IMmotion010 trial,^5^ in which adjuvant atezolizumab did not affect disease-free survival in intermediate-to-high-risk resected or stage M1 cancer in patients with no evidence of disease. Updated results from the KEYNOTE-564 trial^6^ support the use of pembrolizumab vs placebo in the adjuvant setting due to a disease-free survival advantage (HR, 0.63).

Study limitations were use of cumulative rather than individual patient data and lack of direct comparisons between anti–PD-1 and anti–PD-L1 agents from RCTs on RCC. Therefore, only speculative analyses were feasible, although head-to-head studies are warranted.

## Notes

Supplement 1. **eFigure.** PRISMA Flow Diagram for Selection Process

Supplement 2.
Data Sharing Statement
