# Supplementary material for: Comparison of Anti–Programmed Cell Death Ligand 1 Therapy Combinations vs Sunitinib for Metastatic Renal Cell Carcinoma: A Meta-analysis
Source: JAMA Netw Open. 2023 May 18;6(5):e2314144. doi: 10.1001/jamanetworkopen.2023.14144 (PMC10196874; doi:10.1001/jamanetworkopen.2023.14144)
Supplement: Supplement 1. — eFigure. PRISMA Flow Diagram for Selection Process [file jamanetwopen-e2314144-s001.pdf]

Supplementary Online Content

Maiorano BA, Ciardiello D, Maiello E, Roviello G. Comparison of anti–programmed cell death ligand 1 therapy combinations vs sunitinib for metastatic renal cell carcinoma: a meta-analysis. *JAMA Netw Open*. 2023;6(5):e2314144. doi:10.1001/jamanetworkopen.2023.14144

eFigure. PRISMA Flow Diagram for Selection Process

This supplementary material has been provided by the authors to give readers additional information about their work.

© 2023 Maiorano BA et al. *JAMA Network Open.*

eFigure. PRISMA Flow Diagram for Selection Process

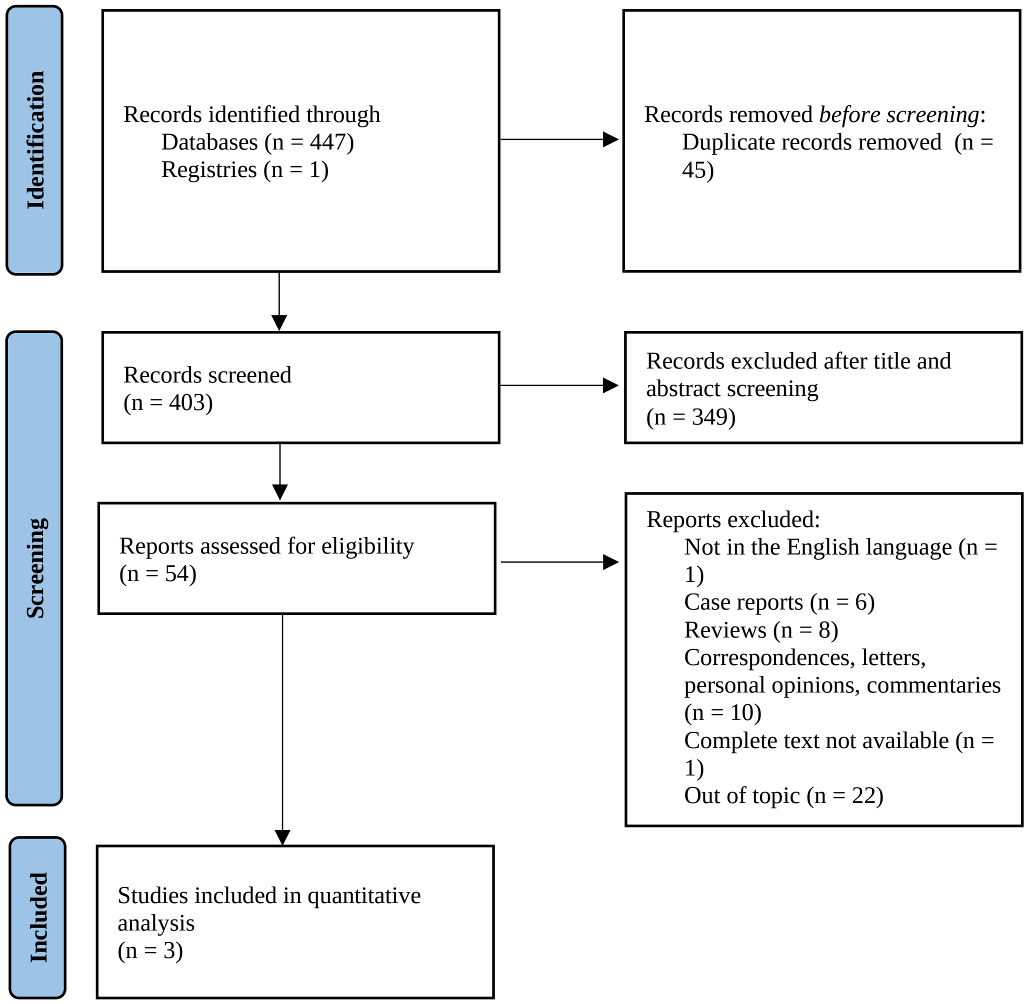

© 2023 Maiorano BA et al. *JAMA Network Open.*
