# Supplementary material for: Comparison of Anti–Programmed Cell Death Ligand 1 Therapy Combinations vs Sunitinib for Metastatic Renal Cell Carcinoma: A Meta-analysis
Source: JAMA Netw Open. 2023 May 18;6(5):e2314144. doi: 10.1001/jamanetworkopen.2023.14144 (PMC10196874; doi:10.1001/jamanetworkopen.2023.14144)
Supplement: Supplement 2. — Data Sharing Statement [file jamanetwopen-e2314144-s002.pdf]

Data Sharing Statement

Maiorano. Comparison of Anti–Programmed Cell Death Ligand 1 Therapy Combinations vs Sunitinib for Metastatic Renal Cell Carcinoma. *JAMA Netw Open*. Published May 18, 2023. doi:10.1001/jamanetworkopen.2023.14144

Data
Data available: No

Additional Information
Explanation for why data not available: Data of meta-analysis derive from published randomized clinical trials
